# Psychiatric Risk in Chronic Spontaneous Urticaria: A Retrospective Cohort Study

**DOI:** 10.2340/actadv.v106.adv-2026-0643

**Published:** 2026-07-06

**Authors:** Philip Curman, Henning Olbrich, Khalaf Kridin, Diamant Thaçi, Ralf J. Ludwig

**Affiliations:** 1 Dermato-Venereology Clinic, Karolinska University Hospital, Stockholm, Sweden; 2 Dermatology and Venereology Division, Department of Medicine (Solna), Karolinska Institutet, Stockholm, Sweden; 3 Department of Medical Epidemiology and Biostatistics, Karolinska Institutet, Stockholm, Sweden; 4 Lübeck Institute of Experimental Dermatology, University of Lübeck, Lübeck, Germany; 5 Department of Dermatology, University-Hospital Schleswig-Holstein (UKSH), Lübeck, Germany; 6 Unit of Dermatology and Skin Research Laboratory, Galilee Medical Center, Nahariya, Israel; 7 Azrieli Faculty of Medicine, Bar-Ilan University, Safed, Israel; 8 Institute and Comprehensive Center for Inflammation Medicine, University of Lübeck, Lübeck, Germany

**Keywords:** urticaria, chronic spontaneous urticaria, depression, stress, TriNetX

## Abstract

Chronic spontaneous urticaria (CSU) has been associated with psychiatric comorbidity, but previous studies have often been limited by cross-sectional designs, reverse causation and surveillance bias. We conducted a retrospective matched cohort study using the TriNetX US Collaborative Network to evaluate the risk of incident psychiatric disorders after CSU diagnosis. Adults with newly diagnosed CSU and no prior psychiatric diagnosis were propensity-score matched 1 : 1 to non-CSU controls. Outcomes were depression, reaction to severe stress and adjustment disorders, schizophrenia and suicidal ideation or suicide attempts during 3 years of follow-up. After matching, 98,785 individuals were included in each group. CSU was associated with a modestly increased risk of reaction to severe stress and adjustment disorders (hazard ratio 1.15, 95% confidence interval 1.09–1.22), consistent across sensitivity analyses. No consistent increased risk was observed for depression, schizophrenia or suicide-related outcomes. A complementary asso-ciation analysis including patients with pre-existing psychiatric disease reproduced previously reported associations with depression and stress-related disorders. These findings suggest that CSU is not broadly associated with incident psychiatric disease but is linked to a small, reproducible increase in stress-related psychiatric morbidity.

**SIGNIFICANCE**

Patients with chronic spontaneous urticaria often experience substantial distress, and previous studies have suggested links with several psychiatric disorders. However, it has been unclear whether these disorders develop after urticaria or mainly reflect pre-existing comorbidity. In this large US-based study, chronic spontaneous urticaria was not clearly associated with new-onset depression, schizophrenia or suicide-related outcomes. A small but consistent increase was observed for stress-related disorders. These findings may help reassure patients while supporting clinical attention to stress-related symptoms when they are present.

Chronic spontaneous urticaria (CSU) is a common inflammatory skin disease characterized by recurrent wheals and/or angioedema without an identifiable external trigger, persisting for more than 6 weeks. Affecting approximately 0.5% to 1% of the global population, CSU is often associated with impaired quality of life, sleep disturbance and substantial healthcare use (1, 2). As such, CSU contributes considerably to the global burden of disease, globally accounting for approximately 4 million disability-adjusted life years (3). In particular, many patients with CSU report emotional distress or coexisting psychiatric symptoms, raising awareness for a possible link between CSU and psychiatric comorbidity (4). In line, several observational studies and meta-analyses have demonstrated an association of CSU with psychiatric diseases, in particular depression, anxiety and stress-related disorders (5–7).

In some studies, the prevalence of depression and anxiety in CSU patients has been reported as high as 25%, especially among those with more severe or poorly controlled disease (5, 8–10). Mendelian randomization (MR) analyses have yielded conflicting results: one study reported that genetic susceptibility to idiopathic urticaria increased the risk of anxiety and major depressive disorder (9), while another found no evidence of a causal relationship (11). These discrepancies may reflect limited phenotypic resolution in urticaria GWAS, which rarely distinguish between disease subtypes (12). Despite this uncertainty, findings have prompted recommendations for psychiatric screening and multidisciplinary care in CSU (1).

Related to reported RWD on the putative asso-ciation with CSU and psychiatric diseases, additional uncertainties remain: Many studies were of a cross- sectional design, lacked temporally defined outcomes or relied on self-reported symptoms, not allowing to determine the directionality of associations. Reverse causation, surveillance bias and residual confounding remain plausible alternative explanations. For instance, patients with undiagnosed psychiatric illness may seek more frequent medical attention, increasing the likelihood of a CSU diagnosis, or conversely, patients with CSU may undergo more frequent health evaluations, leading to earlier detection of psychiatric disorders. Moreover, genetic studies estimate lifelong liability rather than real-world clinical trajectories and may not reflect the onset sequence of diseases (13).

We conducted a large retrospective cohort study using RWD from TriNetX, focusing on the temporal risk of psychiatric disease following CSU diagnosis.

## MATERIALS AND METHODS

### Study design and database

**Fig. 1.**
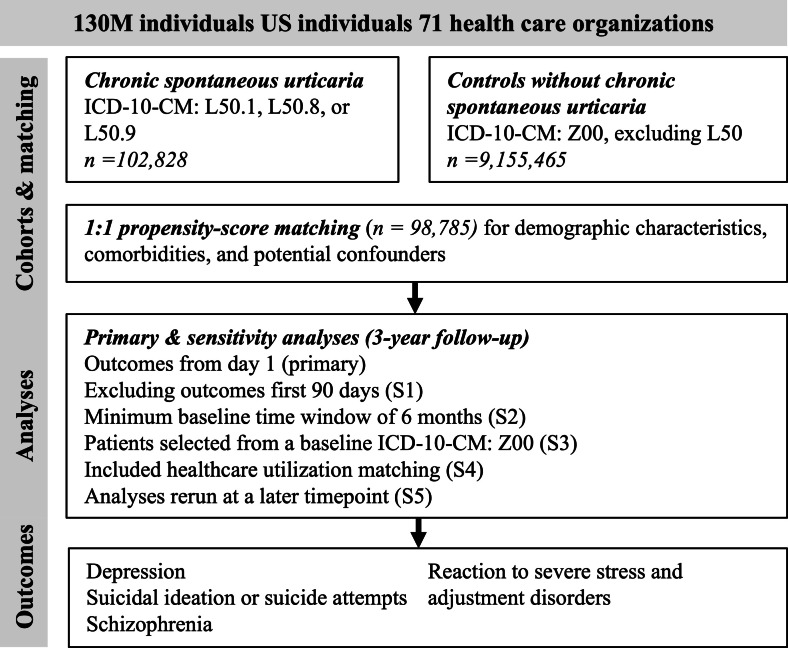
Study design.

A propensity-score-matched (PSM) retrospective cohort study was conducted using the TriNetX healthcare record (EHR) US Collaborative Network, drawing from established protocols (14–16). This network was selected due to the large number of included EHRs, the high degree of covariate documentation and the good representation of the US healthcare-seeking population (17, 18). To address the relationship between CSU and psychiatric disease, we conducted 2 complementary analyses: The temporally anchored analysis evaluated the risk of incident psychiatric disorders following CSU diagnosis using Kaplan–Meier analysis, excluding patients with any prior documentation of the outcomes of interest to ensure temporal validity. The association analysis repeated the same approach without excluding patients with pre-existing psychiatric diseases, thereby capturing the overall comorbidity pattern between CSU and psychiatric disease. Cohort definitions, covariates and outcome measures were defined prior to data extraction (Fig. 1). The study was conducted in accordance with the STROBE guidelines (19).

### Study population

Data for this study were retrieved and analysed from September to October 2025. At the time of analysis, the database provided access to EHRs from over 130.7 million patients across 71 healthcare organizations. Cases and controls were defined by use International Classification of Diseases, 10th Revision, Clinical Modification (ICD-10-CM) codes. Cases with CSU were identified using a temporally anchored definition: Adults (≥18 years) with a diagnostic code for unspecified (L50.9), idiopathic (L50.1), or other urticaria (L50.8), followed by a second code for urticaria (L50) at least 42 days later, to ensure chronicity. To exclude EHRs with any prior documentation of psychiatric disease, a nested cohort definition was used. This excluded patients with any psychiatric diagnosis recorded before the CSU index date, including depression (F32, F33), stress-related or anxiety disorders (F41, F43, F40–F48), schizophrenia (F20), suicidal ideation or attempt (R45.851, T14.91), bipolar disorder (F31), borderline personality disorder (F60.3), substance use disorders (F10–F19) or attention-deficit/hyperactivity disorder (F90.0). The index event for CSU cases was defined as the first documentation of a qualifying urticaria code. Non-CSU controls were identified using a similar definition: Eligible patients were required to have an encounter coded for general medical examination (Z00) without any diagnosis of urticaria (L50) at any time. To ensure data continuity, at least one additional visit was required ≥42 days after the initial encounter. The index event for controls was defined as the first qualifying general examination encounter. Psychiatric exclusion criteria were applied identically to cases.

### Covariates

To minimize bias from confounding, 1 : 1 PSM was used based on a predefined covariate matrix com-prising demographic variables and clinically relevant comorbidities. Demographic covariates included age at index (continuous), sex (female, binary) and self-reported Black or African American ethnicity (binary). Clinical and psychosocial covariates included potential health hazards related to socioeconomic and psychosocial circumstances (ICD-10-CM: Z55–Z65), family history of mental and behavioural disorders (Z81), sleep disorders (G47) and sleep disorders not due to a substance or known physiological condition (F51). All covariates were coded as binary indicators except for age.

### Outcomes

Outcomes were psychiatric diseases, defined by ICD-10-CM codes: Depression (F32), recurrent major depressive disorder (F33), suicidal ideation (R45.851), suicide attempt (T14.91), schizophrenia (F20) and stress-related disorders (F43). F43 was selected as the primary stress-related outcome in preference to broader anxiety disorder categories (F40–F41), which were considered more susceptible to surveillance bias and symptom overlap with CSU-related distress. To assess design specificity and detect residual confounding, we included 2 control outcomes: Systemic antihistamine exposure (positive control), identified using ATC codes R06A, R06 or the Veterans Affairs classification AH000; and varicose veins (negative control), defined by varicose veins of the lower extremities (I83). All outcomes were assessed from the index date until the end of follow-up.

### Primary and sensitivity analyses

The primary analysis investigated outcomes from 1 day to 3 years after the index event. To test the robustness of our findings and address potential sources of bias, we conducted 5 prespecified sensitivity analyses. S1 excluded the first 90 days of follow-up after the index event to minimize the risk of reverse causation, thereby reducing the likelihood that early psychiatric diagnoses reflected pre-existing conditions rather than new-onset disease. S2 mandated a 6-month minimum baseline observation window prior to the index date to ensure sufficient data availability for PSM and to address temporal instability in the electronic health records. S3 retrieved the CSU cohort from among EHRs who had a general health examination (Z00) code, in order to reduce selection bias and better align baseline healthcare behaviour between cases and controls. S4 adjusted for healthcare encounters 1–3 years after the index event to account for surveillance bias arising from differential healthcare utilization. Finally, S5 repeated the primary analysis at a later timepoint since TriNetX is a continuously updated, live database.

In addition to the temporally anchored primary analysis, we performed a complementary association analysis without excluding patients with pre-existing psychiatric diseases. This analysis captured overall comorbidity patterns between CSU and psychiatric diseases, irrespective of disease temporality. The same matching strategy and outcome definitions of the primary analysis were applied. Although assessing psychiatric disease as the index event and CSU as the outcome would theoretically be better suited to examine bidirectionality, this approach was not feasible within TriNetX.

### Statistical analysis

A propensity score for each patient was generated using logistic regression (with exposure as the dependent variable), implemented via the scikit-learn package in Python. Patients were matched 1 : 1 using a greedy nearest neighbour algorithm with a caliper of 0.1 pooled standard deviations of the logit of the propensity score. Baseline characteristics were re-evaluated after matching; continuous variables were compared using the *t*-test, and binary or categorical variables using the *z*-test. Relative risks and risk differences (RDs) were calculated. Time-to-event data were analysed using the Kaplan–Meier method (survival package v3.2–3 in R, R Foundation for Statistical Computing, Vienna, Austria) and validated against outputs from SAS version 9.4 (SAS Institute, Cary, NC). Kaplan–Meier curves were compared using the log-rank test. To quantify effect sizes, hazard ratios (HRs) with 95% confidence intervals (CIs) were derived from univariate Cox regression models. The proportional hazards assumption was assessed using the coxph function in R to ensure interpretability of HRs over time. All outcome events occurring before the index date were excluded from analysis. To account for multiple testing across six predefined outcomes (including the positive and negative control outcomes), a Bonferroni correction was applied, setting the significance threshold at α=0.0083.

## RESULTS

### Study populations

For the temporally anchored analysis, a total of 102,828 EHRs with a documented CSU and 9,155,465 non-CSU controls were identified before matching. After PSM, 98,785 patients were included in each group. Baseline characteristics were well balanced across cohorts post-matching, with standardized differences mostly below 0.0001 for all variables (Table I). The median follow-up was 1,095 days (IQR 625 for CSU; 606 for non-CSU controls). For the association analysis, 178,170 CSU cases and 12,700,065 non-CSU controls were identified. Following PSM, 172,492 patients remained in each group. Covariate balance was achieved across all included variables, with standardized differences<0.001 post-matching. Median follow-up was 1,095 days (IQR 610 for CSU; 600 for non-CSU controls, Table SI).

**Table I.** Baseline characteristics of patients with chronic spontaneous urticaria (CSU) and matched non-CSU controls before and after propensity score matching (PSM)

	Before matching			After matching		
	CSU	non-CSU	Std diff.	CSU	non-CSU	Std diff.
*n*	102,828	9,155,465	–	98,785	98,785	–
Follow-up (days, median, interquartile range)	1,095 (625)	1,095 (612)	–	1,095 (625)	1,095 (606)	–
Age at index (years, mean±SD)	46.5±16.8	48.9±17.1	0.142	46.5±16.8	46.5±16.8	<0.001
Female sex (%)	71.9	53.1	0.395	71.9	71.9	<0.001
Black or African American (%)	14.5	12.5	0.058	14.5	14.5	<0.001
Persons with potential health hazards related to socioeconomic and psychosocial circumstances (%)	1.7	1.0	0.06	1.7	1.7	<0.001
Family history of mental and behavioural disorders (%)	0.1	0.1	0.004	0.1	0.1	0.002
Sleep disorders (%)	12.3	7.5	0.161	12.3	12.3	<0.001
Sleep disorders not due to a substance or known physiological condition (%)	1.7	0.8	0.08	1.7	1.7	<0.001

Data were derived from the TriNetX US Collaborative Network and include adult patients with newly diagnosed CSU and no prior psychiatric diagnoses. Matching was performed 1:1 using propensity scores to balance key demographic and clinical variables. The table reports sample size (n), median follow-up time (with interquartile range, IQR), age at index (mean ± standard deviation, SD), and the proportion of individuals with selected baseline characteristics. Standardized differences (Std diff.) were calculated to assess covariate balance before and after matching; values <0.1 indicate adequate balance. After matching, all variables achieved excellent covariate balance (Std diff. <0.001), indicating successful matching across cohorts.

### Increased risk for stress-related disorders, but not for the other investigated psychiatric outcomes in patients with CSU

**Fig. 2.**
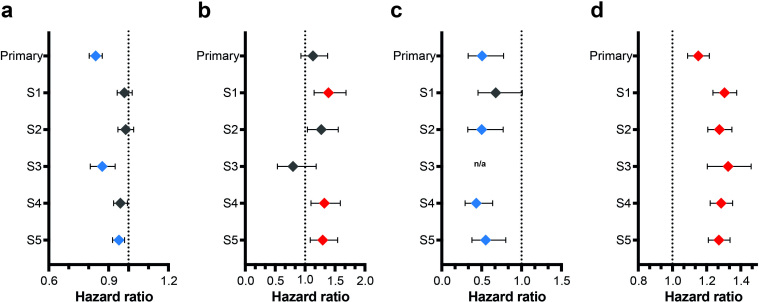
Risk of psychiatric disorders in patients with chronic spontaneous urticaria (CSU) compared to non-CSU controls. The figure presents hazard ratios (HR) with 95% confidence intervals (CI) for the risk of (a) depression, (b) suicidal ideation or suicide attempts, (c) schizophrenia and (d) reaction to severe stress and adjustment disorders in patients with CSU compared to propensity score-matched non-CSU controls. Data were derived from the TriNetX US Collaborative Network. The primary analysis and five sensitivity analyses (S1–S5) were conducted and time-to-event analysis with Kaplan–Meier estimates. While the overall risk for depression, suicide-related outcomes, and schizophrenia was comparable between groups, patients with CSU showed a consistently increased risk of stress-related disorders across all analyses. Diamonds represent HRs; horizontal bars represent 95% CIs. Red indicates statistically significant increased risk (Bonferroni-adjusted *p*<0.0083); blue indicates significantly reduced risk. Confidence intervals are unadjusted for multiple testing.

In the temporally anchored analysis, CSU was associated with a higher risk of stress-related and adjustment disorders compared with matched controls (2.77% vs 2.44%), corresponding to a hazard ratio (HR) of 1.15 (95% CI 1.09–1.22, *p*<0.0001, Fig. 2). This finding was consistent across all 5 sensitivity analyses, with hazard ratios ranging from 1.27 to 1.33 and all *p*-values remaining below the Bonferroni-corrected threshold (*p*<0.0083). In contrast, there was no consistent evidence of changed risk for the other investigated psychiatric outcomes. The positive control outcome (systemic antihistamine exposure) showed a strong association with CSU (HR 1.71, 95% CI 1.66–1.76, *p*<0.0001), confirming the validity of exposure assignment. The negative control outcome (varicose veins) showed no consistent association (HR 1.27, 95% CI 1.06–1.53, *p*=0.0092, Table SII). Absolute risks for all outcomes and analyses are reported in Table SII.

### CSU is associated with increased stress-disorders and depression, and decreased schizophrenia prevalence

In the association analysis, which did not exclude patients with pre-existing psychiatric disease, patients with CSU exhibited a higher prevalence of 2 of the 4 psychiatric diseases compared with matched controls: Reaction to severe stress and adjustment disorders were observed in 7.16% of CSU cases versus 5.31% of controls, corresponding to an odds ratio (OR) of 1.38 (95% CI 1.34–1.41, *p*<0.0001). Depression was documented in 18.79% of CSU cases compared with 17.11% of controls (OR 1.12, 95% CI 1.10–1.14, *p*<0.0001). Schizophrenia was less frequently documented in the CSU group (0.25%) than among controls (0.50%), yielding a significantly lower odds ratio (OR 0.50, 95% CI 0.44–0.56, *p*<0.0001). There was no significant difference in suicide-related outcomes (0.83% in CSU vs 0.89% in controls; OR 0.93, 95% CI 0.87–1.00, *p*=0.057, Table SIII). These results suggest a higher burden of certain psychiatric comorbidities, particularly stress-related conditions and depression, among patients with CSU.

## DISCUSSION

In this large, retrospective cohort study, we investigated the relationship between CSU and psychiatric morbidity. The temporally anchored analysis, designed to estimate incident psychiatric risk in patients free of prior psychiatric disease, demonstrated that CSU is associated with an increased risk for stress-related diseases, whereas no consistent risk changes were observed for depression, suicide-related outcomes or schizophrenia. In a complementary association analysis capturing the broader comorbidity burden irrespective of disease sequence, CSU was associated with a higher prevalence of both stress-related disorders and depression. The discrepancy between these analyses suggests that much of the previously reported comorbidity reflects pre-existing psychiatric vulnerability rather than a consequence of CSU itself. Notably, a lower prevalence of schizophrenia was observed in the association analysis, the interpretation of which remains uncertain and may reflect reduced healthcare engagement, residual confounding or coding-related factors.

For several noncommunicable chronic inflammatory skin diseases, an association with and/or increased risk for psychiatric diseases is documented (20–23). Regarding CSU, studies in diverse populations reported increased psychiatric comorbidity, in particular anxiety and depression (5, 8–10). However, these studies were based on cross-sectional or case-control designs, not allowing to establish a temporal sequence of this association. So far, 2 studies implemented a temporally anchored design: Based on data from Taiwan’s National Health Insurance Research Database, the risk for psychiatric diseases in CSU patients was higher compared to the general population (24). Another study using the same EHR network reported a markedly increased risk for suicide-related outcomes in patients with CSU (15). That analysis was primarily designed to assess all-cause mortality and therefore applied PSM based on covariates relevant to mortality risk. Psychiatric outcomes were assessed using TriNetX’s default outcome exclusion logic, which removes patients with the outcome prior to the index event after matching. While this approach ensures temporality, it can introduce bias by breaking covariate balance for the outcome of interest. In contrast, our present study excluded patients with pre-existing psychiatric diagnoses before matching and implemented a temporally anchored design tailored specifically to psychiatric outcomes. By incorporating psychiatric and healthcare utilization covariates in matching and conducting multiple sensitivity analyses, our approach minimizes the risk of surveillance bias and reverse causation. These refinements likely explain the more conservative effect estimates observed in our analysis, particularly regarding suicide-related outcomes. In summary, no prior study has established a rigorous temporal association between CSU and psychiatric disease, and the only longitudinal analysis available is potentially limited by confounding due to the lack of matching and exclusion of outcomes prior to CSU.

Recent MR studies have attempted to address these limitations by using genetic instruments to assess causality. However, findings remain conflicting. One MR analysis reported that genetic susceptibility to idiopathic urticaria increased the risk of anxiety and that allergic urticaria increased the risk of major depressive disorder (11). In contrast, another MR study found no evidence of a causal relationship in either direction between genetic liability for major psychiatric diseases and urticaria (9). These discrepancies may reflect a fundamental limitation of MR in this context because most GWAS for urticaria do not differentiate between subtypes (12), potentially introducing substantial phenotype heterogeneity. In summary, while RWD analyses to date have been potentially biased by reverse causation and surveillance bias, MR studies may be limited by the absence of robust, well-phenotyped GWAS for CSU.

Our finding of an elevated risk for reaction to severe stress and adjustment disorders aligns with evidence linking CSU to heightened psychological distress and impaired quality of life (4, 25, 26). While earlier studies reported elevated rates of depression and anxiety in patients with CSU, our association analysis confirmed a modestly increased prevalence of depression. The temporally anchored cohort analysis documented an increased risk for stress-related diseases, but no consistent increase in the risk of depression. This discrepancy may be due to differences in methodology, specifically our exclusion of patients with prior psychiatric disease to ensure temporal clarity and minimize reverse causation. Although effect sizes were modest, the consistently stronger associations across sensitivity analyses (HRs 1.27–1.33 vs 1.15 in the primary analysis) suggest the latter may underestimate the true association due to reverse causation or healthcare utilization biases.

Key strengths of this study include its large sample size, use of a federated EHR network, and application of a temporally anchored cohort design. The use of 1 : 1 propensity-score matching and multiple sensitivity analyses strengthen the robustness and validity of our findings. By excluding patients with prior psychiatric diagnoses, we addressed concerns of reverse causation and temporal ambiguity, enhancing the interpretability of risk estimates.

Nonetheless, several limitations warrant careful consideration. First, misclassification bias is possible, given reliance on diagnostic codes and the heterogeneity of EHR documentation. Second, while PSM helped reduce confounding, residual confounding from unmeasured variables may remain. Third, our negative control outcome (varicose veins) yielded a nominally significant hazard ratio of 1.27, which did not survive Bonferroni correction and lacks any plausible biological relationship to CSU. We interpret this as a signal for cautious effect-size interpretation rather than evidence of systematic confounding. Fourth, our case definition relied on repeated urticaria coding separated by at least 42 days to infer chronicity, and while this approach reflects standard real-world coding practice for CSU, the codes used cannot reliably exclude chronic inducible urticarias, which might have different psychosocial consequences, and findings should be interpreted as applicable to a broader chronic urticaria population rather than strictly defined CSU. Fifth, although sensitivity analyses addressed several potential biases (e.g. reverse causation, surveillance bias, healthcare utilization), we could not test for bidirectionality by evaluating psychiatric disease as an index event due to database constraints. Still, healthcare-seeking behaviour may represent an important residual confounder not fully addressed by our healthcare utilization sensitivity analysis; individuals with greater health vigilance or anxiety-related traits may be more likely both to seek care for urticaria symptoms and to receive psychiatric diagnoses. Sixth, generalizability may be limited to the US healthcare-seeking population represented in TriNetX, which may not reflect patterns in under-resourced settings or countries with different healthcare structures. Finally, anxiety disorders (F40–F41) were not evaluated as outcomes. Given the prominence of anxiety in the existing CSU literature, this represents a limitation. Anxiety diagnoses in EHR data are particularly susceptible to surveillance bias due to differential healthcare utilization, and symptom overlap between anxiety and the subjective burden of CSU makes causal interpretation challenging. Future studies incorporating objective measures of healthcare utilization or prospective designs may be better suited to address this question.

Despite these limitations, and in the current absence of controlled prospective investigations, our study offers interim evidence that CSU is not broadly associated with psychiatric risk. Rather, it highlights a modest but reproducible increase in stress-related outcomes. This information may help clinicians reassure patients while remaining attentive to emerging psychosocial symptoms. Specifically, whilst psychological distress is commonly encountered in chronic urticaria, our findings do not support routine psychodermatology referral based on CSU diagnosis alone; rather, referral and multidisciplinary care decisions should be guided by the presence of clinical symptoms.

## Data Availability

Data cannot be made openly available because they are held in a federated de-identified EHR network; access is subject to institutional agreement/licensing with TriNetX.
